# *Ttc21b* is required for proper proliferation of neural progenitor cells

**DOI:** 10.1242/dmm.052392

**Published:** 2026-02-04

**Authors:** Rebekah Niewoehner, David Paulding, Jesus M. Leal, Rebekah Rushforth, Rolf W. Stottmann

**Affiliations:** ^1^ Steve and Cindy Rasmussen Institute for Genomic Medicine, Abigail Wexner Research Institute, Nationwide Children's Hospital, Columbus, OH 43205, USA; ^2^ Division of Human Genetics, Cincinnati Children's Hospital Medical Center, Cincinnati, OH 45215, USA; ^3^ Department of Pediatrics, The Ohio State University College of Medicine, Columbus, OH 43210, USA

**Keywords:** Ttc21b, Microcephaly, Neurogenesis, Mouse, Forebrain

## Abstract

Primary cilia play a pivotal role in cellular signaling and development. Human primary microcephaly is strongly associated with pathogenic variants in primary cilia genes. Here, we examine the role of *Ttc21b*, a component of the intraflagellar transport-A complex, during mouse forebrain development by using a *Ttc21b^alien^* null allele. Our findings reveal that significant microcephaly in homozygous mutants is caused by disrupted neural progenitor proliferation and differentiation. Histological and immunohistochemical analyses show an enlarged ventricular zone and reduced cortical plate thickness accompanied by altered mitotic spindle angles, suggesting defects in symmetric versus asymmetric cell divisions. Embryonic expression patterns suggest that perdurant TTC21B protein underlies these phenotypes. Progenitor proliferation kinetics were disrupted along with changes in TBR2-positive intermediate progenitors and TBR1-positive early-developing neurons. Neuronal processes in the cortical plate were significantly shortened. Our findings support a model in which early expression of *Ttc21b* in neural precursor cells destined for the forebrain is critical to ensure TTC21B protein levels to sustain subsequent neural progenitor proliferation and differentiation. These results advance our understanding of the role primary cilia have in cortical development.

## Teaser

**Summary:** Loss of *Ttc21b* in mouse leads to changes in early neurogenic cell division patterns. We also discuss why previous forebrain-specific deletions of *Ttc21b* fail to recapitulate the microcephaly phenotype seen in the null allele.

## INTRODUCTION

The primary cilium is a microtubule-based extension of the cell found on virtually every cell type. This organelle, once considered a vestigial structure (Alberts et al., 1994), is now known to be crucial for proper signal transduction in several contexts. Primary cilia are supported by the axonemal microtubules anchored to the basal body/centriole. Several different signal transduction effector molecules require trafficking through the cilium mediated by evolutionarily conserved intraflagellar transport (IFT) proteins. The most notable of these are arguably proteins of the GLI family, transcription factors required for hedgehog signaling. Kinesin motors move IFT-B complexes and associated cargo along the microtubules of the axoneme towards the distal tip of the cilia in an anterograde manner, and dynein motors move IFT-A complexes and cargo in a retrograde fashion back towards the cell body. IFT-A transport is also essential for organizing material at the base of cilia, especially G-protein-coupled receptors (GPCRs) (Baker and Beales, 2009; Goetz and Anderson, 2010; Reiter and Leroux, 2017; Wheway et al., 2018). In mammalian systems, the primary cilium is required for proper regulation of multiple signaling pathways inside the cell, including the Hedgehog, polycystin, Notch, BMP, PDGF, GPCR, Hippo, mTor, canonical Wnt, and planar cell polarity pathways (Goetz and Anderson, 2010; Wheway et al., 2018). Primary cilia have been previously shown to be crucial for forebrain development in multiple contexts (Zaidi et al., 2022).

Primary microcephaly is a congenital malformation of cortical development with known genetic and environmental causes (Phan and Holland, 2021). There are currently approximately thirty genes firmly associated with microcephaly according to the most recent annotations in the Online Mendelian Inheritance in Man (OMIM) database. Half of these genes are known to have roles in primary cilia biology, with a third already well known to be important for primary cilia form and/or function, and another five with emerging evidence to be considered ciliary genes. Some of these ciliary genes include CDK5RAP2, CENPJ and WDR62 (Barrera et al., 2010; Ding et al., 2019; Zhang et al., 2019). Many mouse models with deletions of primary cilia genes also have microcephaly and other forebrain phenotypes (Broix et al., 2018; Ding et al., 2019; Zhang et al., 2019; Shohayeb et al., 2020). However, the effects of these ciliary perturbations can be quite varied. It is now clear that ciliary signaling is an important regulator of mammalian brain growth, but the molecular mechanisms are not fully elucidated.

Tetratricopeptide repeat domain 21B (TTC21B, also known as IFT138, THM1) is a component of the intraflagellar transport-A complex. Human conditions associated with variants in *TTC21B* include nephronopthesis (MIM# 613820) and short-rib thoracic dysplasia 4 with or without polydactyly (MIM# 613819). Other associations have been made with kidney disease (Bullich et al., 2017), heterotaxy (Strong et al., 2021) and retinopathy (Ben-Yosef et al., 2021). In addition to these monogenic presentations, there is significant evidence in humans and model organisms that *Ttc21b* can genetically interact with other ciliopathy genes to create a wide spectrum of ciliopathy phenotypes including Jeune asphyxiating thoracic dysplasia (Davis et al., 2011). A null allele of *Ttc21b* recovered from a mouse N-ethyl-N-nitrosourea (ENU) mutagenesis forward genetic screen has multiple embryonic phenotypes consistent with ciliopathies (Tran et al., 2008). We have previously shown that a null allele of *Ttc21b* in the mouse leads to a smaller forebrain and an anterior−posterior patterning phenotype in the early developing anterior nervous system (Stottmann et al., 2009). These forebrain phenotypes have not yet been identified in patients with *TTC21B* variants. The severity of the *Ttc21b-*null (*Ttc21b^aln/aln^*) mice suggests that any humans identified to date with *TTC21B* pathogenic variants only have hypomorphic alleles, and that alleles severe enough to recapitulate the mouse microcephaly may compromise other organ systems enough to prevent survival to term. Alternatively, because the current human genetic findings in patients with *TTC21B* variants do not include brain malformations, there may be an understandable reticence to make new associations with *TTC21B* and microcephaly in emerging human cases. Taken together, it is clear that primary cilia biology is important for mammalian brain development and that *Ttc21b*, in particular, has a role which is not fully understood. Further study of these phenotypes will help fully elucidate the role of primary cilia in neural development.

We have previously attempted to use conditional deletion of *Ttc21b* in the mouse developing forebrain to study the molecular basis of this microcephaly in the absence of any confounding effects of the patterning deficit (Snedeker et al., 2017). Much to our surprise, deletion of *Ttc21b*, by using the established *Foxg1-Cre* and *Emx1-Cre* tools that are well-known to be active in the early neuroepithelium (Hebert and McConnell, 2000; Gorski et al., 2002), did not recapitulate the microcephaly phenotype seen in the germline null animals (Snedeker et al., 2017). The control of neural progenitor proliferation is thought to be autonomous to the forebrain tissue, making these phenotypes particularly intriguing. We have continued to study the processes leading to microcephaly in the *Ttc21b* mouse mutants to elaborate on the role of primary cilia in neural progenitors and forebrain development.

## RESULTS

### *Ttc21b^aln/aln^* brains are significantly smaller with disruptions to the proliferative ventricular zone

**Fig. 1.**
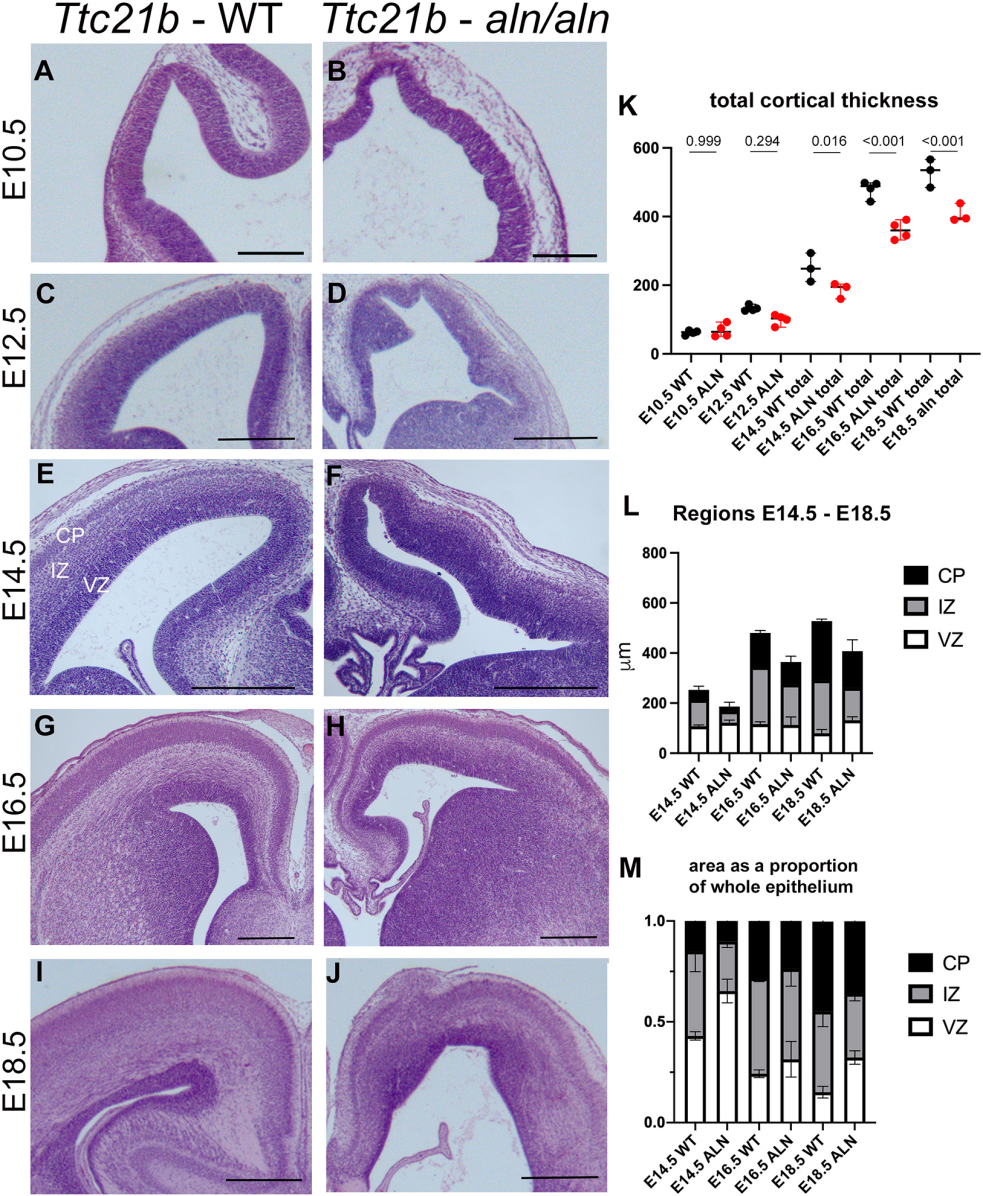
**Histological analysis of *Ttc21b^aln/aln^* cortical development.** Sections of wild-type (A,C,E,G,I) and *Ttc21b^aln/aln^* (B,D,F,H,J) brains at E10.5 (A,B), E12.5 (C,D), E14.5 (E,F), E16.5 (G,H), and E18.5 (I,J). Total cortical thickness is quantified (K, *t*-test *P* values shown) and shows robust reduction in mutants by E14.5. Width of the ventricular zone (VZ), intermediate zone (IZ) and cortical plate (CP) are compared between wild-type (WT) and *Ttc21b^aln/aln^* (ALN) brains (L) and shown as proportions of total width (M). Scale bars: 500 µm (A-F), 1 mm (G-J). *n*=4 (E10.5, E12.5, E16.5) or *n*=3 (E14.5, E18.5) embryos per genotype. Data shown in K are the median±95% confidence interval, L and M are the average±s.e.m.

Previous work has shown that conditional deletion of *Ttc21b* from the forebrain did not result in the smaller brain size observed in *Ttc21b^alien/alien^* homozygous mutants (Snedeker et al., 2017). In order to understand the mechanisms leading to such a reduction, we returned to analyze the null allele. We have previously shown that *Ttc21b^alien/alien^* homozygous mutants on the Friend leukemia virus B NIH Jackson (FVB/NJ) genetic background have an even more severe phenotype than those maintained on a largely C57BL/6J (B6) background (Snedeker et al., 2019). However, other previous work from our group has shown that congenic FVB mice lead to high rates of exencephaly and/or embryonic death preventing an efficient study of forebrain development. We, therefore, performed this current study only on embryos derived from intercrosses between *Ttc21b^alien/wt^* heterozygous carriers maintained on an FVB genetic background and those maintained on a C57BL/6J (B6) background, i.e. F1 hybrids. We have previously shown that the forebrains of *Ttc21b^alien/alien^* mice are appreciably smaller as early as embryonic day (E) 12.5 (Stottmann et al., 2009, Fig. 1). Here, we examined the cortical epithelium from E10.5 through E18.5. Histological features of *Ttc21b^alien/alien^* mutants included an irregular edge to the epithelium in mutants at E10.5 (Fig. 1A,B). The size of the proliferative ventricular zone appeared enlarged as soon as it was a distinctive layer within the cortex, while the cortical plate housing differentiated neurons appeared smaller (Fig. 1F,H,J). We noticed that the differences between the mutants described here are somewhat variable, both within a single mutant brain and throughout the mutants studied as a whole. We quantified the width of the cortex at E10.5 through E18.5, as well as the ventricular zone, intermediate zone and cortical plate individually between E14.5 and E18.5. While obviously dysmorphic and irregular at E10.5, the total cortical thickness of mutants was significantly reduced by E14.5 and only further deviates from wild type over time (Fig. 1K). While the whole cortex was thinner, the relative proportion of the area taken up by the ventricular zone was greater at all stages examined and the cortical plate was always smaller (Fig. 1L,M).

### Ttc21b expression is not high in forebrain

**Fig. 2.**
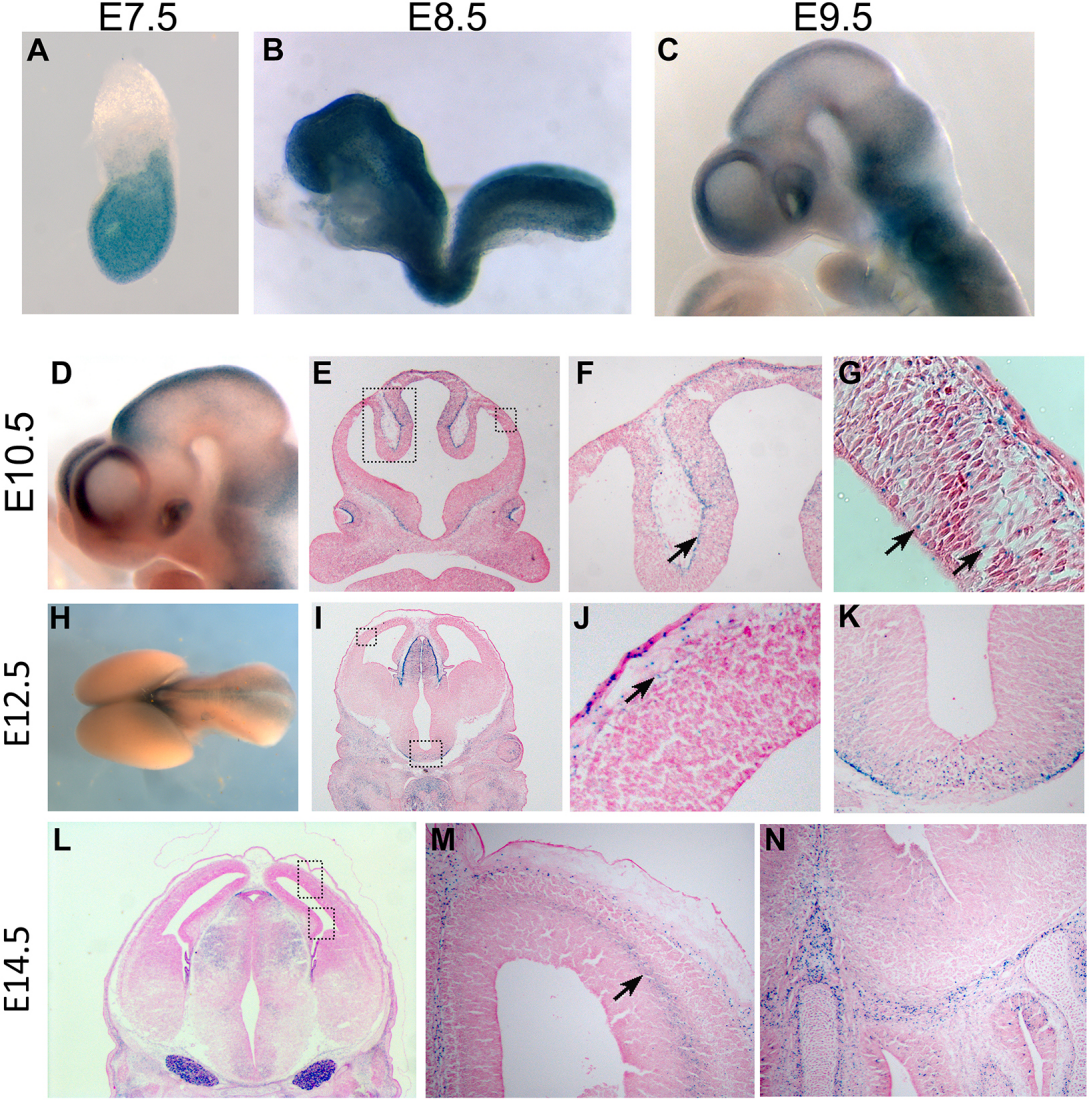
***Ttc21b* lacZ expression.** (A-N) *Ttc21b^tm1a^* embryos were stained with X-gal to highlight *Ttc21b* expression in whole-mount embryos (A-D,H) or after sectioning (E-G,I-N) at embryonic days as indicated. Boxed areas in E are shown magnified in F and G, boxed areas in I and L are shown magnified in J and M. Arrows indicate specific areas of lacZ expression.

The surprising results from the genetic deletions of *Ttc21b* from the forebrain (Snedeker et al., 2017) motivated us to take a much closer look at the expression of *Ttc21b* throughout forebrain development. We used the lacZ expression from the *Ttc21b^tm1a^* gene trap allele to visualize high expression throughout the embryo at E7.5 (Fig. 2A) and E8.5 (Fig. 2B). By E9.5 (Fig. 2C) and E10.5 (Fig. 2D), whole-mount analysis clearly showed that *Ttc21b* expression becomes much more restricted, but that the dorsal forebrain is an area that appears to retain some lacZ signal. Histological section analysis showed the expression is most prominent in the layer immediately adjacent to the neural epithelium (Fig. 2E,F, arrow in F), which is most likely the neural-crest-derived meningeal layer. Close examination did reveal scattered lacZ-positive cells within the forebrain neural epithelium, but we saw no clear pattern to these cells (Fig. 2G, see arrows). Whole-mount analysis at E12.5 indicated even lower *Ttc21b* expression (Fig. 2H) and sections again showed very few positive cells scattered throughout the epithelium. We noted they are, again, much more obvious in the layer of cells immediately adjacent to the epithelium (Fig. 2I-K). Sections at E14.5 showed this pattern continues but we also noted a new population of cells in the intermediate zone (Fig. 2M, arrow). Given the previously demonstrated role of *Arl13b* in tangential migration (Higginbotham et al., 2012) and the position of these cells, we hypothesize these are interneurons migrating from the ventral brain structures towards their final destination in the cortical plate. We noted interesting expression in increasingly later stages of brain development, but these are not immediately relevant to the rest of the work presented here.

These findings, further exploring the expression patterns of *Ttc21b*, may explain our previous results, in which *Foxg1-Cre* and *Emx1-Cre* did not recapitulate the *Ttc21b^aln^*germ line microcephaly phenotype (Snedeker et al., 2017). These Cre transgenes drive Cre recombination in the neural epithelium, which is clearly not a region of high *Ttc21b* expression (Hebert and McConnell, 2000; Gorski et al., 2002). Rather, the highest levels of *Ttc21b* gene expression were at earlier stages in tissues that, in turn will give rise to the cortical epithelium. We, therefore, propose a model in which the high levels of *Ttc21b* gene expression ‘load’ those cells destined to make up the forebrain with TTC21B protein. Thus, we propose that TTC21B protein is present long after gene expression is diminished and that is an especially perdurant protein compared to average protein half-lives (Belle et al., 2006; Price et al., 2010; Cambridge et al., 2011).

### Neural progenitor proliferation and differentiation are altered in *Ttc21b^aln/aln^* mutants

**Fig. 3.**
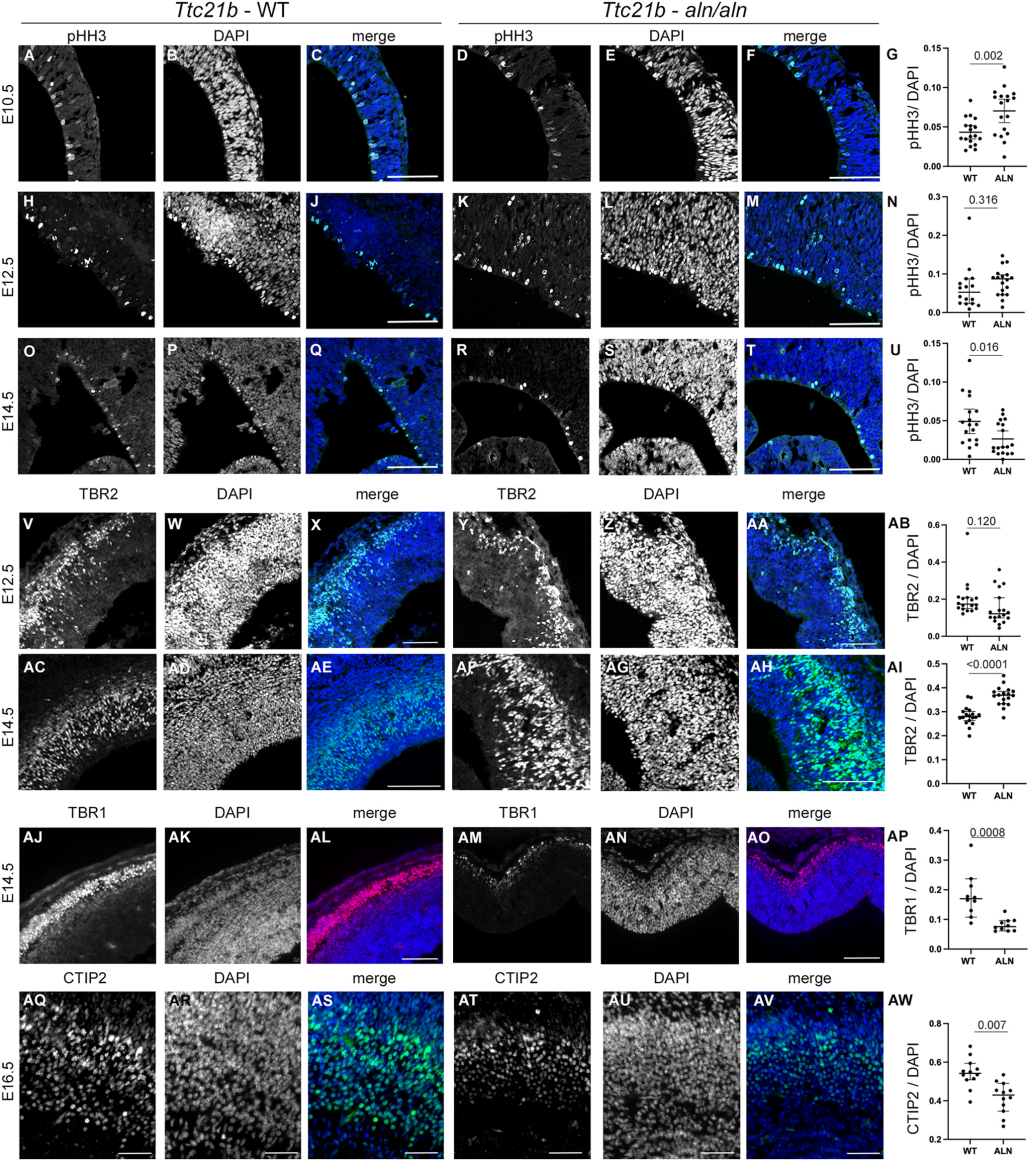
**Neurogenesis in *Ttc21b^aln/aln^* cortical development.** Immunohistochemistry for neuronal proliferation and differentiation markers was performed for pHH3 (A-U), TBR2 (V-AI), TBR1 (AJ-AP), and CTIP2 (AQ-AW). Proliferation is marked by pHH3 (A,D,H,K,O,R) at E10.5 in wild type and *Ttc21b^aln/aln^* mutants at E10.5 (A-F), E12.5 (H-M) and E14.5 (Q-T). Quantification of mitotic indices are shown for each age in G,N, U with *t*-test *P* values. TBR2-positive (V,Y,AC, AF) intermediate progenitors are shown at E12.5 (V-AA) and E14.5 (AC-AM) and quantified in AB, AI. TBR1-positive cells (AJ, AM) shown at E14.5 and quantified in AP. CTIP2-positive cells (AQ, AT) are reduced in mutants at E16.5 and quantified in AW. Scale bars: 100 µm (A-AO), 50 µm (AQ-AV). *n*=3−6 sections from each of three embryos per each genotype. Quantification data shown are the median±95% confidence interval. Data plotted by animal littermates is shown in Fig. S1.

We measured rates of progenitor proliferation by using immunohistochemistry for the mitotic marker pHH3 at multiple stages of forebrain development in *Ttc21b^aln/aln^* mutant mice. We noted an increased proportion of cells positive for pHH3 at E10.5 (Fig. 3A-G, *P*=0.002). At E12.5, we saw a much smaller change in the mitotic index (Fig. 3H-N, *P*=0.316) and by E14.5, we noted a marked decrease in the number of mitotic cells (Fig. 3O-U, *P*=0.016). These data were consistent with the histological analysis we present, where the size of the proliferative ventricular zone was increased in mutants relative to wild type at early stages in neurogenesis (Fig. 1). The TBR2-positive intermediate progenitors are also a critical population for generating the proper brain size and are the result of asymmetric divisions of apical progenitors. We noted a small decrease in the relative number of TBR2-positive cells at E12.5 (Fig. 3V-AB, *P*=0.120) but a marked increase at E14.5 (Fig. 3AC-AI, *P*<0.0001). We also analyzed patterns of cell death but saw no appreciable signal in control or *Ttc21b^aln^* sections (Fig. S1J-L).

In order to assess any effects changes in behavior of these early progenitors might have on later stages of maturation, we observed TBR1-positive cells. TBR1 initially marks postmitotic cells in the preplate layer and is also expressed in the deep layer neurons born from the earliest apical progenitor cells to undergo terminal divisions (Bulfone et al., 1995; Hevner et al., 2001). At E14.5, wild-type brains show a robust layer of TBR1-positve cells close to the pial surface (Fig. 3AJ-AL). These cells were obviously and markedly reduced in the *Ttc21b^aln/aln^* mutants (Fig. 3AM-AP). We saw a similar result when we examined CTIP-2 positive cells just 2 days later (E16.5). These results confirm a reduction in *Ttc21b^aln^* mutant neurogenesis compared to that of controls (Fig. 3AO-AW).

### The mitotic angle of ventricular progenitors is slightly altered in *Ttc21b^aln/aln^* mutants

**Fig. 4.**
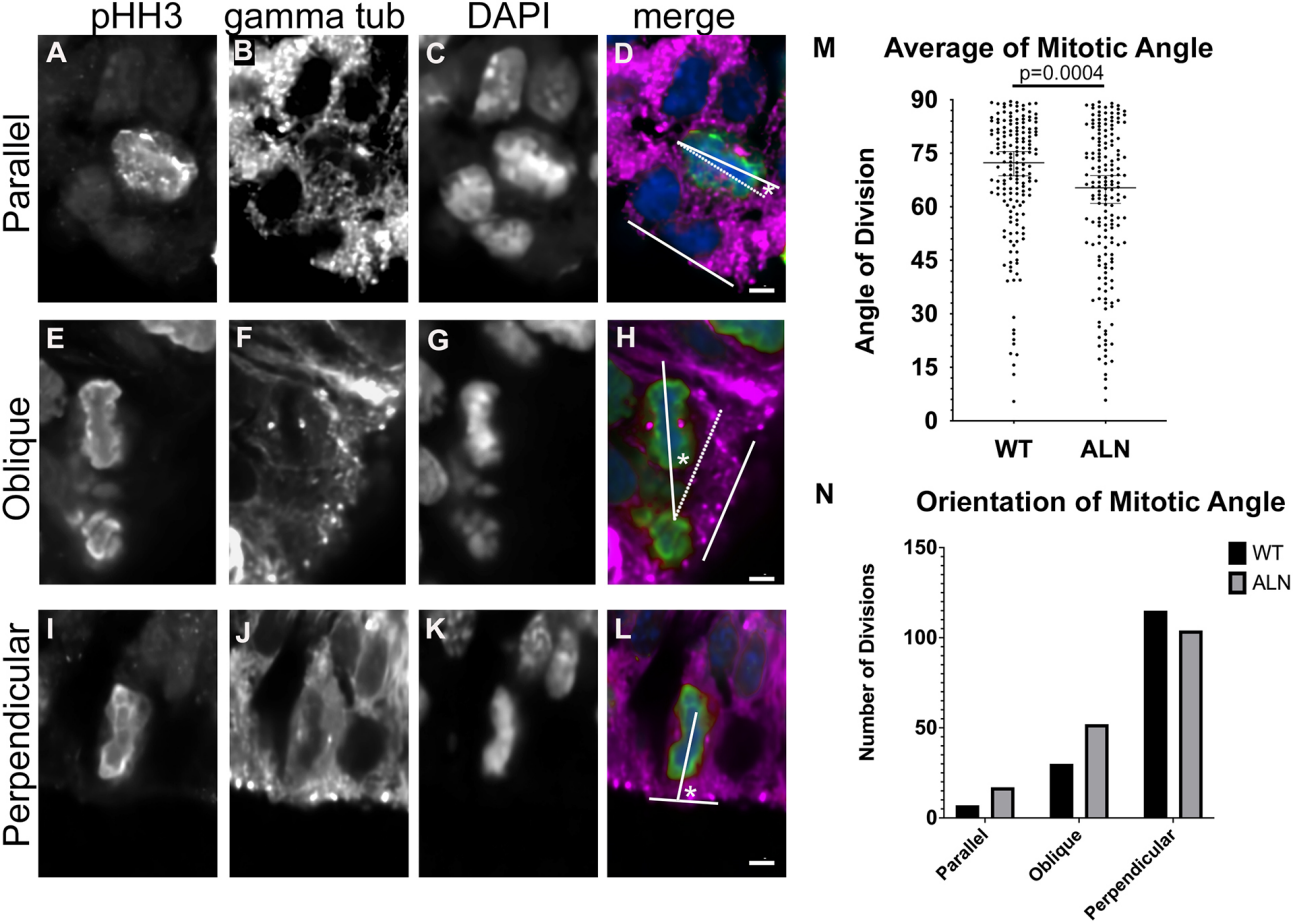
**Mitotic angle of neural progenitors in E12.5 *Ttc21b^alien^* brain.** (A-L) The angle of the mitotic plane in dividing neural progenitor cells was determined with the use of immunohistochemistry for pHH3 (A,E,I), gamma tubulin (B,F,J) and DNA (C,G,K), merged images are shown in D, H and L. Dividing cells were identified, one line was drawn identifying the plane of mitotic angle between the two centrosomes and a second line to identify the plane of the ventricular surface. The angle between these two lines is the ‘mitotic angle’. Angles (indicated by asterisks) between 0° and 30° are parallel (D), between 30° and 60° are oblique (H), and between 60° and 90° are perpendicular (L). (M) The mitotic angles in wild-type (WT) and *Ttc21b^aln/aln^* mutant (ALN) mutant neural progenitor cells were quantified and the average for each is shown (Student's *t*-test was used to calculate the *P* value). (N) Graph showing the distribution of parallel, oblique and perpendicular angles in WT and ALN cells. Scale bars: 10 µm. Cells were counted from at least four different sections of at least three embryos of each genotype, and all values are plotted in M.

Given the behaviors we just described, we hypothesized the angle of the neuroprogenitor cell mitotic spindles relative to the plane of the ventricular zone may be different in *Ttc21b^aln/aln^* mutants. This mitotic angle has been previously correlated with the fate of the cells after mitosis (Taverna et al., 2014; Matsuzaki and Shitamukai, 2015). Cells with a plane of mitosis perpendicular to the plane of the ventricular zone are said to undergo a symmetric division with fairly equal division of cellular contents and are more likely to go on to generate two ‘mother’ stem cells (Taverna et al., 2014; Matsuzaki and Shitamukai, 2015). In contrast, division planes more parallel to the ventricular zone are thought to asymmetrically divide cellular contents and the cell closer to the ventricular zone is likely to remain a stem cell while the ‘daughter’ cell will begin to differentiate into a neuron, or a TBR2-positive basal progenitor (Taverna et al., 2014; Matsuzaki and Shitamukai, 2015). We used immunostaining for pHH3 and gamma-tubulin to mark centrosomes for over 150 mitoses in both control and mutant brains from multiple animals at E12.5. We measured the mitotic angle of the neural progenitor cells (Fig. 4D,H,L) and found the *Ttc21b^aln^* mutants had a smaller average angle of mitoses, suggesting mitoses in mutants were, on average, more asymmetric (Fig. 4M). We further subdivided the angle measurements into three categories of parallel mitoses (angles 0−30°, Fig. 4A-D), oblique (30−60°, Fig. 4E-H) and perpendicular (60−90°, Fig. 4I-L). Mutant brains had slightly more parallel and oblique mitoses compared to wild type (Fig. 4N). All of these findings are consistent with the previous data and ultimate histological phenotypes described above. Taken together, our findings suggest that loss of *Ttc21b* leads to changes in centrosome dynamics in the proliferative neural epithelium.

**Fig. 5.**
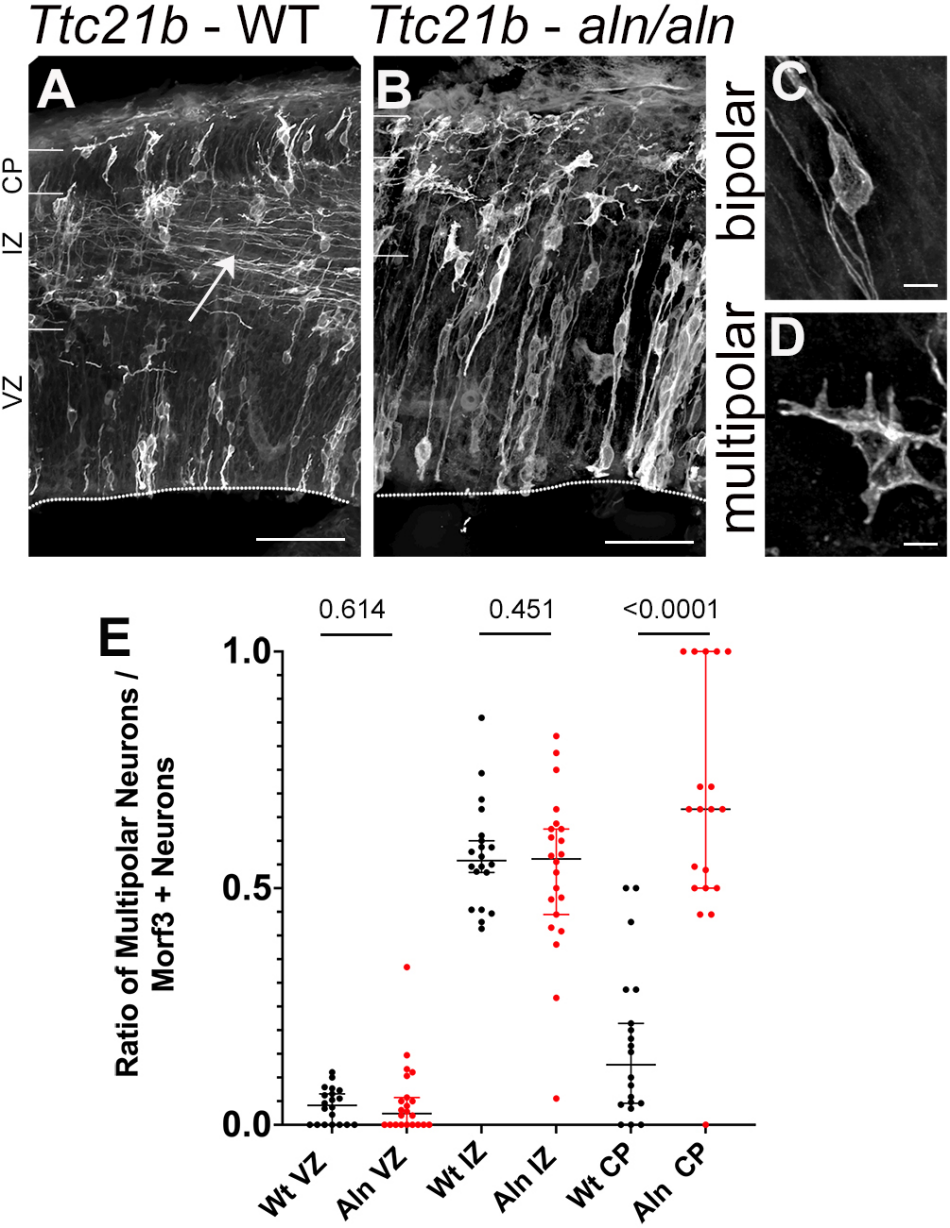
**Cellular morphology in E14.5 *Ttc21b^alien^* neurons.** The MORF3 reporter allele was used with the *Emx1-Cre* line to stochastically label cells in the forebrain. (A,B) Section images from WT (A) and *Ttc21b^aln/aln^* mutant mouse brains (B) show marked differences in cellular extensions made in the dorsal portions of cortex (arrow in A). (C,D) Examples of bipolar (C) and multipolar (D) cells. (E) The ratio of multipolar cells in each region of the brain is plotted for WT and *Ttc21b^aln/aln^* mutant mice. The cortical plate quantification shows that *Ttc21b^aln/aln^* mutant cells are much more likely to retain a multipolar morphology. Scale bars: 50 µm (A,B), 5 µm (C,D). *n*=proportions of all cells clearly visible (∼15−65) in each of approximately four sections (dots in E) from three embryos of each genotype. Data shown are the median±95% confidence interval.

In order to further explore the later stages of cortical development in the *Ttc21b^aln/aln^* mutants with the hypothesis that intracellular cytoskeletal dynamics were perturbed upon loss of *Ttc21b*, we utilized the MORF3 reporter allele, which stochastically labels individual clones within a Cre-positive lineage (Veldman et al., 2020). We used the *Emx1-Cre* to label a small portion of neurons within the developing forebrain in control (*Emx1cre*/wt; *MORF3*/wt;*Ttc21b^wt/wt^*) and mutant (*Emx1cre*/wt; *MORF3*/wt;*Ttc21b^aln/aln^*) embryos. The process of radial migration includes the change in cellular morphology from a multipolar arrangement to a bipolar arrangement as the migrating cell interacts with the radial glial cell scaffold used for the radial migration (Stouffer et al., 2016; Francis and Cappello, 2021). Given the profound morphological deficits we saw in the *Ttc21b^aln/aln^* mutants, we hypothesized these cellular reorganizations would be dramatically affected in the mutants. We quantified the proportion of total cells at E14.5 within the ventricular zone comprising a multipolar morphology (as compared to bipolar) and did not see a difference between wild type and mutant; although, we again noted the dramatically expanded ventricular zone in mutants (Fig. 5E). However, as neurons begin to mature in the dorsal cortex, wild-type tissue showed extensive lateral projections (arrowhead in Fig. 5A) that are almost completely missing in the *Ttc21b^aln/aln^* mutant tissue (Fig. 5B). We also noted a striking change in cellular morphology within the cortical plate, where wild-type cells most often display a bipolar morphology, while mutants cells much more often retain a multipolar morphology (Fig. 5C-E).

## DISCUSSION

Here, we further report on the striking microcephaly seen in the *Ttc21b^aln/aln^* homozygous mouse mutants. We showed that early neural progenitor proliferation kinetics are disrupted upon loss of *Ttc21b* and saw an accompanying shift in the angle of the mitotic spindle relative to the ventricular zone. We also observed an increase in TBR2-positive intermediate progenitors at E14.5 and a reduction in mature TBR1-positive and CTIP2-positive neurons in the developing cortical plate at E14.5 and E16.5. We showed the surprising result that *Ttc21b* expression is not very prominent in the neural epithelium at the stages when these molecular phenotypes are observed. Rather, expression is much higher at earlier stages. We proposed a model, in which TTC21B protein is especially perdurant and the relevant genetic ablations to cause microcephaly are best performed at significantly earlier stages. This would potentially explain why Cre transgenic lines commonly used in the field and expected to affect neural progenitor proliferation, such as *Foxg1-Cre* and *Emx1-Cre* (Hebert and McConnell, 2000; Gorski et al., 2002), did not recapitulate the null phenotype described here and previously reported (Stottmann et al., 2009).

### When and where is Ttc21b required for forebrain development

The recovery of the *alien* missense null allele of *Ttc21b* recovered from an ENU mutagenesis (Tran et al., 2008) and the first follow-up analysis (Stottmann et al., 2009) documented several processes dependent on *Ttc21b*, including brain development. Earlier work indicated patterning deficits as early as the first stages of neurogenesis (Stottmann et al., 2009). This had prompted us to employ a novel conditional allele of *Ttc21b* to ablate the gene in just the developing forebrain with the Emx1-Cre allele to define the role of *Ttc21b* in neurogenesis (Snedeker et al., 2017). When this ablation did not recapitulate the microcephaly, we repeated the experiment with a slightly earlier acting Cre but again failed to replicate the null phenotype. These Cre transgenics are well-established genetic tools to assess molecular signaling in neural progenitor cells. Other *Ttc21b* expression domains at neurogenesis stages (E10.5−14.5) may be the most critical source(s) of *Ttc21b*. We noted expression in the developing meninges (Fig. 2). However, we previously have ablated *Ttc21b* with an AP2-Cre, which is active in the neural and non-neural ectoderm, and only recovered brains of increased size, suggesting no unique trophic source in the meninges. Another possible *Ttc21b* expression domain could create a non-autonomous signaling source from the ventral regions of the brain. This is a valid model that can be genetically addressed (i.e. Dlx5/6 Cre is expressed in ventral regions) but it was difficult for us to generate a convincing model of non-autonomous action from this region of the developing brain. This line of reasoning lead us to implicate the early expression of *Ttc21b*. Early embryonic *Ttc21b* expression at E7.5 and E8.5 is much stronger as measured by the lacZ reporter allele in our hands than the neural tissues. However, testing the model that the relevant *Ttc21b* expression domain is at earlier stages of tissues in cells in younger embryos which are ultimately fated to become the telencephalon, has been unexpectedly challenging. The *Hesx1-Cre* mouse allele which has been shown to stimulate loxP recombination in the anterior neuroectoderm (Andoniadou et al., 2007) and was assumed to be a good candidate for a relevant *Ttc21b* domain, did not lead to robust recombination activity in our hands. We attempted an embryo-wide ablation of *Ttc21b* with an inducible Cre [*Gt(ROSA)26Sor^tm1(cre/ERT2)Tyj^/J*; (Ventura et al., 2007)] but treatment with tamoxifen at early stages (E4.5, E5.5), with a dose high enough to stimulate broad recombination, led to significant embryonic death, precluding efficient measurements of brain size at late organogenesis stages. Direct measurement of TTC21B protein is hampered by current unavailability of robust antibodies. We constructed an epitope-tagged allele of *Ttc21b* as an alternative tool but this did not lead to effective translation of an epitope-conjugated form TTC21B for reasons we were unable to determine. In future studies, further testing of this model will necessitate the use of additional Cre transgenic mouse lines and/or a new allele of *Ttc21b* with a degron tag to allow controlled degradation of endogenous protein (Phanindhar and Mishra, 2023). Thus, there is not yet a clear understanding of the source of the most critical TTC21B protein to regulate neurogenesis. We anticipate addressing this in the future by using further genetic ablations.

### Primary cilia and the cell cycle in the developing forebrain

Cell proliferation defects in cilia mutants, such as those highlight here, are consistent with previous studies, as assembly and disassembly of the cilium is necessary to allow the centrosome to be used in the mitotic apparatus. Several excellent reviews comprehensively discuss findings in this area, and we would particularly call attention to those by Hasenpusch-Theil and Theil (2021), Liu et al. (2021) and Zaidi et al. (2022).

The increased rate of mitosis at E10.5 (Fig. 3) is likely the result of increased numbers of symmetric divisions (Fig. 4). As these are, in turn, more likely to generate more stem cells, this could very well explain the increased thickness of the ventricular zone we noted as well as the physical irregularities seen in the ventricular surface (Fig. 1). Alterations in this process will have cascading effects through the remainder of corticogenesis.

### Ttc21b mutants have decreased numbers of mature neurons

The data we presented here, showing increased apical progenitor proliferation at E10.5, increased TBR2-positive basal progenitors at E14.5 and a slight shift in the mitotic angle towards parallel divisions at E12.5, are consistent with increased rates of indirect neurogenesis as compared to direct neurogenesis from the division of a ‘mother’ cell directly into two ‘daughter’ cells. We further showed reductions in the number of both TBR1- and CTIP2-positive neurons in the mutants. Primary cilium proteins have previously been implicated in this choice between the two possible cell fates, although changes in ciliary dynamics have now been shown to both promote and inhibit direct neurogenesis. Furthermore, several previously published mouse mutants have microcephaly or macrocephaly, depending on which ciliary gene(s) are altered and in which spatiotemporal domain (Wilson et al., 2012; Foerster et al., 2017; Snedeker et al., 2017; Hasenpusch-Theil and Theil, 2021).

Beyond the initial perturbations of neural progenitor proliferation and differentiation, we hypothesized the dysmorphic cortex in the *Ttc21b^aln/aln^* homozygous mutants, along with previous evidence for a role of cilia in neuronal migration (Higginbotham et al., 2012), to be the result of altered radial migration due to an inability of the cell to properly employ cytoskeletal elements. We used the MORF3 reporter allele to highlight cellular shape but did not see evidence of defect transition from multipolar to bipolar morphology as neurons utilize the radial glial scaffolds for radial migration. However, we did see a marked decreased in cellular extensions as cells do reach their final destinations. Given that only some processes mediated by the cytoskeleton seem to be disrupted in *Ttc21b^aln/aln^* mutants, this may be a fruitful avenue for further investigation. We continue to demonstrate that cilia do, indeed, have “multiple and varied roles in cortical development” (Hasenpusch-Theil and Theil, 2021). However, deeper understanding of this organelle in this tissue will only lead to better comprehension of human malformations of cortical development, especially microcephaly.

## MATERIALS AND METHODS

### Mouse husbandry

All animals were maintained through a protocol approved by Nationwide Children's Hospital Medical Center IACUC committee (IACUC2021-AR2100067). Mice were housed with a 12-h light cycle with food and water *ad libitum*. Mouse euthanasia was performed in a carbon dioxide chamber, followed by secondary cervical dislocation. Genotyping was performed via PCR and gel electrophoresis on a 2% agarose gel or custom Taqman assays. All mouse alleles have been previously published. *Ttc21b^aln^* (MGI:2181876) can be obtained from the authors upon satisfaction of institutional sharing requirements. The conditional gene trap allele of *Ttc21b*, C57BL/6N-*Ttc21b*^*tm2a(KOMP)Wtsi/MbpMmucd*^ (RRID:MMRRC_050240-UCD), is available from the Mouse Mutant Resource and Research Centers (MMRRC). The Emx1-Cre allele B6.129S2-*Emx1*^*tm1(cre)Krj*^/J (RRID:IMSR_Jax:005628), the MORF3 reporter C57BL/6-*Gt(ROSA)*^26Sortm3(CAG-sfGFP*)Xwy^/J (RRID:IMSR_Jax:035403) and the tamoxifen-inducible Cre allele B6.129-*Gt(ROSA)26Sor*^*tm1(cre/ERT2)Tyj*^/J (RRID:IMSR_Jax:008463) are available from The Jackson Laboratory. Whole-brain and skeletal images were taken on a Zeiss Discovery V12 microscope (Zeiss, St. Louis, MO).

### Histology

Brains were dissected, fixed in formalin for 48 h, washed in 70% ethanol, and then dehydrated and paraffin-embedded by the morphology core facility. Embedded brains were sectioned on a microtome at 10 µm (Sakura, Hayward, CA). Sections were placed on glass slides (Cardinal Health, Dublin, OH), baked for >1 h at 37°C, and stained with hematoxylin and eosin using standard methods. Body and brain weights were measured using a standard chemical scale. For cortical and cerebellar measurements ZEN 3.7 software was used, with area measured in µm^2^ and length in µm. A minimum of three animals from at least two distinct litters were measured for each genotype. Embryos were stained for lacZ protein expression using standard protocols (Behringer, 2014).

### Anatomical measurements

Matched brain sections from control and mutant embryos were chosen to be compared in anterior−posterior position at E10.5, E12.5, E14.5, E16.5 and E18.5. The cortical thickness was measured at the central-most point of the right dorsal pallium of the cortex. Measurements were taken using ImageJ for total cortical thickness, averaged, and compared for controls and mutants of all stages. For the embryonic stages E14.5, E16.5 and E18.5, the cortex was divided into ventricular zone, intermediate zone and cortical plate. This separation was determined by examining morphology by eye and was kept consistent across all samples.

### Immunohistochemistry

After dissection, brains were fixed for 1−2 days in PFA at 4°C. PFA was replaced with 30% sucrose for 2 days before brains were embedded in Optimal Cutting Temperature solution (Sakura) and stored at −80°C. 10-µm sections were obtained for mouse brain and human organoid samples by using a Leica CM 1860 cryostat, placed on glass slides and stored at −20°C. Slides selected for immunohistochemistry (IHC) were pre-warmed at 42°C for 10−15 min. Antibody retrieval was performed as previously described (Bittermann et al., 2019). The following antibodies were used: anti-pHH3 (Sigma Aldrich AB_477043, 1:500, RRID:AB_477043), anti-TBR1 (Abcam ab31940, 1:200, RRID:AB_2200219), anti-TBR2 (Abcam ab23345, 1:200, RRID:AB_778267), anti-CC3 (Cell Signaling #9661, 1:300, RRID:AB_2341188), anti-gamma tubulin (Sigma T6557, 1:1000, RRID:AB_477584), anti-CTIP2 (1:300, Abcam ab28448, RRID:AB_1140055), Alexa Fluor 488 goat anti-rabbit (Invitrogen A11008, 1:500, RRID:AB_143165), Alexa Fluor 488 goat anti-mouse (Invitrogen A11001, 1:500, RRID:AB_2534069), Alexa Fluor 555 goat anti-rabbit (Invitrogen A21428, 1:500, RRID:AB_2873183), Alexa Fluor 610 goat anti-rabbit (Invitrogen A20980, 1:500, RRID:AB_1500648), Alexa Fluor 555 goat anti-mouse (Invitrogen A21422, 1:500, RRID:AB_141822). Slides were mounted in Prolong Gold Antifade medium (Invitrogen P36935). The MORF3 allele allows staining with a V5 antibody (Invitrogen R960-25, 1:1000,RRID:AB_2556564). All antibodies are established commercially available reagents and validations were performed by manufacturer.

### Cell counting

Cells positive for CC3, PHH3, TBR2, TBR1 and CTIP2 were counted in the NIS-Elements Analysis program (Nikon, Melville NY) by manually drawing a region of interest (ROI) across the cortex from the ventricular zone to the pial surface and counting immuno-positive cells and DAPI positive cells using a bright spot detection function. Cells counts were normalized to total DAPI cells present in the ROI.

### MORF3 visualization

Brains were sectioned 40 µm on a Leica CM 1860 cryostat, placed on glass slides, and stored at −20°C. Slides selected for IHC were pre-warmed at 42°C for 10−15 min. Antibody retrieval was performed as previously described (Bittermann et al., 2019). Confocal imaging for the MORF3 allele was done with a Nikon AX R Confocal microscope and analyzed using NIS Elements. For the quantification of neuronal morphology in the cortex, neurons with three or more processes were considered multipolar and neurons with two processes were considered bipolar. The data are shown as the proportion of multipolar cells over the whole number of MORF-3 cells analyzed.

### Statistical analysis

Data plots and subsequent analyses were performed with Prism 9 (GraphPad, San Diego, CA). A Student's *t*-test was performed for experiments with two groups. An ANOVA with Tukey's multiple comparison tests was performed for experiments with more than two comparisons. ANOVA *P*-values are usually stated in the text or figure legend and specific relevant *P*-values for the multiple comparisons are shown in the relevant figure. We report the statistical test values directly rather than assigning a significance symbol to provide all the data for the reader. Data shown are the median±95% confidence interval. No data were excluded from the analysis. Sample sizes were consistent with other experiments in the literature which have been adequately powered. In general, experimentalists were blinded to genotype of specimens when quantifying results of immunohistochemistry.

## Supplementary Material

10.1242/dmm.052392_sup1 Supplementary information
