# Supplementary material for: Ttc21b is required for proper proliferation of neural progenitor cells
Source: Dis Model Mech. 2026 Feb 4;19(1):dmm052392. doi: 10.1242/dmm.052392 (PMC12919951; doi:10.1242/dmm.052392)
Supplement: Supplementary information [file dmm-19-052392-s1.pdf]

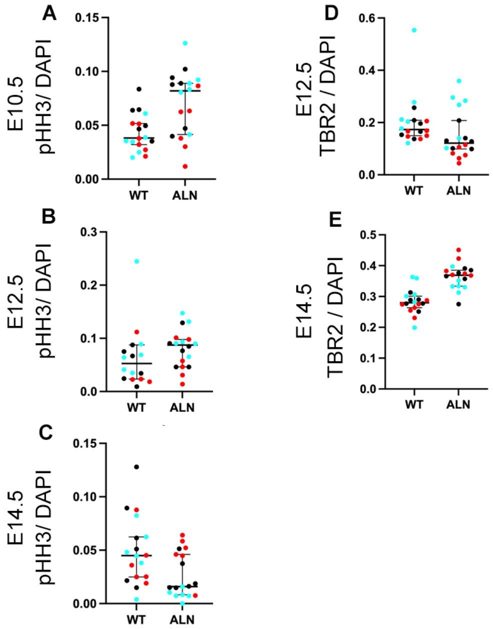

Fig. S1. Neurogenesis in *Ttc21b^{alien}* cortical development. (A-I) Quantifications shown in Figure 3 G, N, U, AB, AI are shown with the different colors indicating which values from specific sections come from which individual embryos. (t-test p values shown)
The order of graphs shown in Figure 3 is repeated here.

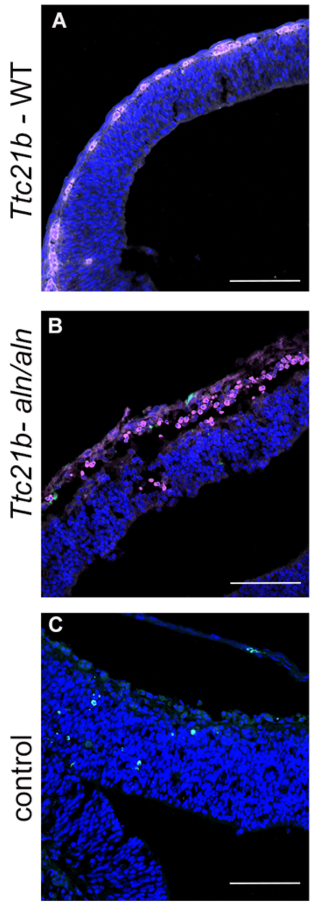

Fig. S2. Apoptosis is not evident in *Ttc21b*^{alien} mutant brain. Cleaved-caspase 3 (green) immunohistochemistry was used to assess cellular apoptosis in wild-type (A)and *Ttc21b*$^{aln/aln}$ mutants (B) with no appreciable signal seen in each. The red/magena signal is used to indicate the levels of auto fluorescence to disinguish from green CC3-positive cells. A positive control from a different genotype (*Tubb2b*^{brdp}, (Stottmann et al. 2013)) was used to validate the CC3 antibody (green cells in C are consistent with previous data showing apoptotic cells in this mutant). Scale bars = 100 µm.
